# Retention and critical outcomes among new methadone maintenance patients following extended take-home reforms: a retrospective observational cohort study

**DOI:** 10.1016/j.lana.2023.100636

**Published:** 2023-12-04

**Authors:** Arthur Robin Williams, Noa Krawczyk, Mei-Chen Hu, Lexa Harpel, Nicole Aydinoglo, Magdalena Cerda, John Rotrosen, Edward V. Nunes

**Affiliations:** a Columbia University Department of Psychiatry, New York, USA; b New York State Psychiatric Institute, New York, USA; c Department of Population Health, NYU Grossman School of Medicine, USA

**Keywords:** Opioid use disorder, Methadone, Opioid treatment programs, COVID-19

## Abstract

**Background:**

Approximately 1800 opioid treatment programs (OTPs) in the US dispense methadone to upwards of 400,000 patients with opioid use disorder (OUD) annually, operating under longstanding highly restrictive guidelines. OTPs were granted novel flexibilities beginning March 15, 2020, allowing for reduced visit frequency and extended take-home doses to minimize COVID exposure with great variation across states and sites. We sought to use electronic health records to compare retention in treatment, opioid use, and adverse events among patients newly entering methadone maintenance in the post-reform period in comparison with year-ago, unexposed, controls.

**Methods:**

Retrospective observational cohort study across 9 OTPs, geographically dispersed, in the National Institute of Drug Abuse (NIDA) Clinical Trials Network. Newly enrolled patients between April 15 and October 14, 2020 (post-COVID, reform period) v. March 15-September 14, 2019 (pre-COVID, control period) were assessed. The primary outcome was 6-month retention. Secondary outcomes were opioid use and adverse events including emergency department visits, hospitalizations, and overdose.

**Findings:**

821 individuals were newly admitted in the post-COVID and year-ago control periods, average age of 38.3 (SD 11.1), 58.9% male. The only difference across pre- and post-reform groups was the prevalence of psychostimulant use disorder (25.7% vs 32.9%, p = 0.02). Retention was non-inferior (60.0% vs 60.1%) as were hazards of adverse events in the aggregate (*X*^2^ (1) = 0.55, p = 0.46) in the post-COVID period. However, rates of month-level opioid use were higher among post-COVID intakes compared to pre-COVID controls (64.8% vs 51.1%, p < 0.001). Moderator analyses accounting for stimulant use and site-level variation in take-home schedules did not change findings.

**Interpretation:**

Policies allowing for extended take-home schedules were not associated with worse retention or adverse events despite slightly elevated rates of measured opioid use while in care. Relaxed guidelines were not associated with measurable increased harms and findings could inform future studies with prospective trials.

**Funding:**

10.13039/100000016USDHHS10.13039/100000026NIDA10.13039/100015615CTNUG1DA013035-15.

**Research in context**

**Evidence before this study**

Models for methadone maintenance treatment for opioid use disorder vary worldwide. Despite longstanding pharmacy-based dispensing models in some Western nations, the United States has required patients receiving methadone maintenance to receive in-person dosing at specialised Opioid Treatment Programs (OTPs) for the first 90 days of care with allowances for only a single take-home dose at a given point in time in the second 90 days of care, typically restricted to patients with total cessation of drug use. Following COVID-19 onset in March 2020, the US changed policy to allow for take-homes of up to 28 days for new patients at OTPs if sufficiently stable, creating a natural experiment to study the impact of less restrictive regulations early in care.

We searched PubMed using the following search terms through February 2023: methadone maintenance, opioid treatment programs, and take-homes. We did not identify prior studies with structured electronic health record data examining extended take-homes in the first months of care with US populations. However, a few qualitative studies of patients' and providers’ perspectives on regulatory reforms have been published. Therefore, this is the first published quantitative study of take-home schedules, adverse events, and clinical outcomes among patients newly entering care at US OTPs.

**Added value of this study**

Treatment outcomes (6-month retention and rates of adverse events) among new methadone maintenance intakes under relaxed regulations were similar to those of the year-ago unexposed control group subjected to daily attendance, despite higher rates of opioid use while in care in the post-COVID period. Results did not change under moderator analyses accounting for stimulant use and site-level variation in take-home schedules.

**Implications of all the available evidence**

Findings suggest that policies allowing for up to 28 days of take-homes early in care, akin to monthly pharmacy-based prescriptions, is not associated with increased dropout or adverse events, even in the presence of elevated rates of opioid use. However, the impact of environmental variables such as COVID-related isolation practices and service disruptions may have impacted patterns of care utilisation and findings should be interpreted with caution.

## Introduction

There are approximately 1800 opioid treatment programs (OTPs, colloquially known as methadone maintenance programs) treating approximately 400,000 unique individuals in the U.S. on a given day.^1^ While only treating a fraction of the estimated 7.6 million individuals with opioid use disorder (OUD),^2^ OTPs have been a mainstay of the addiction treatment architecture, especially in urban areas, as retention rates for methadone are typically higher than with other MOUD modalities (i.e., buprenorphine, or extended-release naltrexone).^3^, ^4^, ^5^, ^6^ OTPs are required by US law to provide an entire array of medical and psychiatric preventative, case management, and treatment services including infection disease testing. Since the inception of methadone maintenance treatment in 1972,^7^ state and federal rules have mandated a minimum frequency of clinic visits, especially in the first 180 days of care.^8^ Given the significant regulatory framework, findings from US-based OTPs may not generalize to less restrictive settings in other nations.

Until recently, federal laws required daily clinic attendance for the first 90 days (generally only permitting a single take-home dose for Sundays or holidays). After this, patients were allowed to have every other day attendance for the following 90 days in care, but this generally applied only to patients who did not miss clinic visits and achieved sustained abstinence from regular opioid use.^9^ Evidence is limited on how these restrictive policies, constraining methadone delivery and take-home doses, impact treatment outcomes. They are known to impose a considerable burden on patients and have the potential to undermine retention in treatment and discourage patients with OUD from seeking care.^10^

Following the onset of the COVID-19 Public Health Emergency in March 2020, the Substance Abuse and Mental Health Services Administration (SAMHSA) announced a “blanket waiver” that allowed, from the outset of treatment, take-home doses for up to 28 days for “stable” patients and for up to 14 days for “somewhat stable” patients.^11^^,^^12^ Given the airborne transmission of COVID, the purpose of the waiver was to allow clinics to reduce patient travel and time spent at clinic which involves close contact with clinic staff and other patients. The initial definitions of “stable” and “somewhat stable” were left intentionally vague to allow sites to be nimble in responding to local conditions and use clinical judgment given the heterogeneity in patient case mix and COVID impacts across OTPs. A given patient's clinical stability was generally assessed by program adherence, time in treatment, patterns of opioid and other drug use (e.g., sedative-hypnotics), and ability to safely store methadone.^9^ Abrupt relaxation of methadone regulations represented a natural experiment to study the impact of reforms.

Since the implementation of these changes, many studies have reported on the feasibility and safety of extended take-home guidelines, and the significant benefits such regulatory changes have had on patient treatment experiences and quality of life.^9^^,^^10^ However, research is lacking on the impact of these policy changes on patient retention in care, long the gold standard of treatment success. The Optimal Policies to Improve Methadone Maintenance Adherence Long-term (OPTIMMAL) Study, conceived in the Spring of 2020, and funded by the National Institute on Drug Abuse Treatment Clinical Trials Network,^13^ was designed to evaluate these questions. The primary outcome was 6-month retention, considered a minimum yet measurable duration of methadone maintenance treatment endorsed by the Centers for Medicaid and Medicare Services.^14^ Secondary outcomes included relevant clinical outcomes: opioid use and critical outcomes including emergency department (ED) visits, hospitalizations, and drug overdose. We hypothesized that outcomes of interest would be equivalent between the 2020 (post-COVID) cohort compared to the 2019 (pre-COVID) cohort of patients newly admitted to methadone maintenance.

## Methods

### Study design

**Fig. 1:**
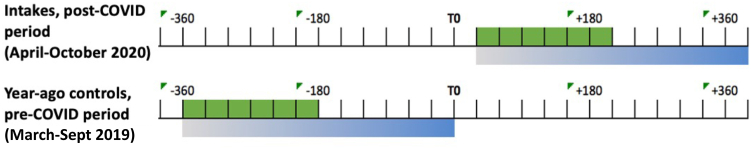
**Study Design Scheme**. T0 indicates the onset of COVID-19 era reforms including the SAMHSA blanket waiver allowing for extended take-homes (March 15, 2020). Green sections indicate sample enrollment periods (6 months each), and blue sections indicate outcome windows (up to 6 months following intake for each patient while remaining in care).

We analyzed outcomes among patients newly admitted for methadone maintenance for up to 6 months post-intake across 9 OTPs in 9 different states. The primary outcome was 6-month retention and secondary outcomes included rates of opioid use and adverse events during care. The exposed group began treatment post-COVID onset between April 15 and October 14, 2020, allowing for a one-month lag following the adoption of the SAMHSA blanket waiver at each site around March 15, 2020. A control group, also followed for up to 6 months post-intake, was selected from the year-ago, unexposed pre-COVID period, in 2019 (Fig. 1).

### Setting

Eligible sites were licensed OTPs that were required to have been continuously in operation since at least January 1, 2018, and have integrated electronic health record (EHR) systems that tracked patient admission data reportable to state regulators, medication dosing tracked on a per-visit basis, and urine drug testing results archived electronically for all patients. As we sought to study the impact of changes in delivery of methadone maintenance in response to the COVID-19 pandemic and revised rules issued by SAMHSA–in particular relaxation of clinic visit frequency and allowing increased methadone take-homes, OTPs must have changed their procedures in accordance with the new SAMHSA allowances, implementing reduced visits and accelerated methadone take homes for appropriate patients. Sites had to maintain clinic visits, methadone dosing and dispensing data, urine toxicology data, and other clinical data in an electronic medical record (EHR) readily accessible for chart review and data abstraction. We intentionally sought to recruit a range of sites that varied in their approach to moving patients toward extended take-homes, less frequent drug testing, and remote visits. OTPs with previous experience successfully participating in research studies were prioritized. Some naturalistic variation in the extent of these changes was expected and seen as a strength of the study design. Site recruitment was conducted through the CTN network. Efforts were made to recruit a range of sites that varied in their implementation of revised practices under COVID-related reforms through a structured site questionnaire inquiring about protocols pre- and post-COVID during the site recruitment process as well as each site's basic patient demographics, typical case mix, and retention metrics. Given that sites were connected to the CTN, and many had prior research experience, they were disproportionately affiliated with academic medical centres and not-for-profit.

### Eligible patients

Eligible patients were considered for inclusion irrespective of age. Patients had to have been enrolled either between April 15 and October 14, 2020 (post-COVID) (Fig. 1), or March 15 to September 14, 2019 (Pre-COVID control group). Starting the exposed cohort in April allowed for a one-month lag following the announcement of the SAMHSA blanket waiver on March 15, 2020 (T_0_) considering that during the first few weeks after reforms were implemented, implementation was likely to be uneven. To be eligible for inclusion, patients had to be confirmed as initiating a new treatment episode of methadone maintenance (i.e., not buprenorphine or naltrexone). Admissions for methadone tapers (i.e., detoxification) were excluded and we employed a 90-day lookback to remove individuals with recent methadone treatment and transfers from other methadone programs in recent months to identify index care episodes. In accordance with applicable federal regulations (45 CFR 46.116(d)), the study was approved for a waiver of informed consent by the New York University Institutional Review Board.

### Data source

Data were manually extracted and coded by OTP-based research assistants and data managers who had direct access to participating site EHR systems. All data were entered into a custom, web-based data management system house at Columbia University which had built-in checks and safeguards to consistently structure data and minimize data entry errors. To assure data integrity and minimize the risk of systemic biases in extraction methods across sites, all sites’ research teams attended training during orientation. Subsequently, a central quality assurance monitor and the OPTIMMAL project coordinator met twice monthly with all sites during data entry to remotely view charts and data entry methods. Additionally, random audits were conducted among the first 10 charts entered and then among all other data thereafter to detect errors and omissions. Quality checks continued after final data entry to identify errors and omissions and sites were able to correct erroneous or missing entries. All data were de-identified prior to analysis.

### Methadone dosing

Due to stringent federal and state regulations, OTPs are required to consistently and completely document all methadone dispensed in the EHR, whether for in-clinic observed dosing or for take-home doses that are ingested off-site at later dates. As a result, all participating OTPs had EHR systems designed to track every dose of methadone dispensed including the total milligrams per dose. While patients remained in care (i.e., had not yet been discontinued), every day in care was coded for either a 1). In-clinic directly observed methadone dose, 2). Take-home methadone dose (for up to 27 days in addition to the in-person dose), or 3). Missed methadone dose (e.g., patient returned to clinic one or more days later than scheduled).

### Outcomes

The primary study outcome was a binary indicator of 6-month retention (yes/no). Discontinuation was defined as a gap of 30+ days without medication, consistent with most states’ requirements for administrative discharge periods, with the final day in care attributed to the last clinical visit irrespective of any take-homes that may have been provided on that date. We additionally studied time-to-discontinuation which was operationalized as days from the beginning of the observation period (date of intake) to the first day of the 30-day or more gap in medication coverage as defined for the primary outcome.

Secondary outcomes included frequency of opioid-positive urines as well as adverse events related to opioid use including ED visits, hospitalizations, overdose, and death. Opioid use was measured as follows: for every 30-day block starting at the beginning of the observation period, the proportion of urines collected during that block that are positive (i.e. for one or more opioid results, not including methadone) was calculated. If no urine drug test results were documented for an individual in a given block, that individual was counted as missing during that block. Adverse events were documented in the EHR progress notes section and categorized as follows: ED visits, hospitalizations, overdose events (non-fatal), or death.

### Covariates

Covariates were selected to adjust for differences between the groups that have been shown in prior studies to be associated with retention in care. Prior studies have found that patient retention in methadone maintenance is linked to a mix of baseline patient characteristics (e.g., female sex, older age, less cocaine and alcohol use), in-treatment variables (e.g., methadone dose and other drug use during care)^15^ and environmental factors (e.g., community beliefs about and attitudes toward methadone, proximity to treatment).^16^, ^17^, ^18^, ^19^, ^20^, ^21^, ^22^ For this study, we systematically captured (Table 1) patient demographic characteristics (age, sex, race, insurance status, justice involvement, marital status, level of education), infectious disease status (HIV and hepatitis C), addiction severity and use history, and comorbidity data at intake including other substance use disorders. These data elements were typically entered into the EHR by OTP staff during a patient's intake visit or early in treatment. However, there was variation across sites, in part due to variation in state-level reporting requirements, in how these baseline characteristics were catalogued and sites may have differed in their ascertainment of sex as a biological variable. Additional baseline variables related to drug use history are presented in Supplementary Table S1.

**Table 1:** Baseline covariates of new intakes at opioid treatment programs, by COVID-19 exposure (N = 821).

	Pre-COVID (n = 386)	Post-COVID (n = 435)
	No. (%)/Mean (SD)	No. (%)/Mean (SD)
Age	39.6 (11.7)	38.8 (11.1)
Age group ≥ 40	165 (42.8)	171 (39.3)
Gender		
Male	236 (61.1)	256 (58.9)
Female	150 (38.9)	179 (41.1)
Race		
White	272 (70.5)	336 (77.2)
Black	50 (12.9)	35 (8.1)
Asian	2 (0.5)	1 (0.2)
Native Hawaiian, Pacific Islander	3 (0.8)	2 (0.5)
American Indian Alaskan Native	10 (2.6)	12 (2.8)
Other (including multi-race)	29 (7.5)	35 (8.0)
Missing	20 (5.2)	14 (3.2)
Ethnicity		
Hispanic	35 (9.1)	48 (11.0)
Non-Hispanic	273 (70.7)	312 (71.7)
Missing	78 (20.2)	75 (17.2)
Health insurance		
None	59 (15.3)	59 (13.6)
Grant Funded	36 (9.3)	33 (7.6)
Medicaid	249 (64.5)	290 (66.7)
Medicare	14 (3.6)	22 (5.1)
Medicaid and Medicare	10 (2.6)	8 (1.8)
Commercial insurance	10 (2.6)	17 (3.9)
Other (including missing)	8 (2.1)	6 (1.4)
Marital status		
Never married	137 (35.5)	160 (36.8)
Married	95 (24.6)	89 (20.5)
Formerly married	64 (16.6)	77 (17.7)
Single (Unknown marital status)	87 (22.5)	98 (22.5)
Missing	3 (0.8)	11 (2.5)
Highest level of education attained		
<8 years	14 (3.6)	25 (5.7)
Some high school	94 (24.4)	95 (21.8)
High school graduate	141 (36.5)	176 (40.5)
Some college	83 (21.5)	97 (22.3)
College graduate	39 (10.1)	30 (6.9)
Missing	15 (3.9)	12 (2.8)
Housing status		
Homeless	61 (15.8)	62 (14.2)
Unstable	80 (20.7)	94 (21.6)
Secure housing	224 (58.0)	256 (58.9)
Other	12 (3.1)	17 (3.9)
Missing	9 (2.3)	6 (1.4)
Employment status		
Full-time	72 (18.7)	83 (19.1)
Part-time	39 (10.1)	35 (8.1)
Retired	6 (1.6)	3 (0.7)
Disabled	46 (11.9)	45 (10.3)
Student	1 (0.3)	1 (0.2)
Unemployed	139 (36.0)	162 (37.2)
Out of label force	76 (19.7)	99 (22.8)
Missing	7 (1.8)	7 (1.6)
Arrest within 30 days		
Yes	13 (3.4)	14 (3.2)
No	223 (57.8)	245 (56.3)
Missing	150 (38.9)	176 (40.5)
Criminal justice involvement		
Yes	265 (68.6)	297 (68.3)
No	89 (23.1)	95 (21.8)
Missing	32 (8.3)	43 (9.9)
HIV		
Yes	13 (3.4)	7 (1.6)
No	193 (50.0)	222 (51.0)
Missing	180 (46.6)	206 (47.4)
Hepatitis C		
Yes	145 (37.6)	155 (35.6)
No	163 (42.3)	199 (45.8)
Onset age of opioid use	24.7 (9.9)	23.6 (8.7)
Onset age of opioid use group		
<18	114 (29.5)	135 (31.0)
18–25	126 (32.6)	150 (34.5)
≥18	130 (33.9)	132 (30.3)
Missing	16 (4.1)	18 (4.1)
Primary route for opioid use		
Oral	29 (7.5)	21 (4.8)
Smoking	8 (2.1)	12 (2.8)
Intranasal	119 (30.8)	127 (29.2)
Injection	212 (54.9)	262 (60.2)
Drug use disorder^a^		
Alcohol	33 (8.6)	38 (8.7)
Opioid	384 (99.5)	432 (99.3)
Cannabis	52 (13.5)	48 (11.0)
Cocaine	49 (12.7)	70 (16.1)
Stimulant	58 (15.0)	82 (18.9)
Psychostimulant NOS	99 (25.7)	143 (32.9)
PCP	2 (0.5)	1 (0.2)
Benzodiazepine	15 (3.9)	15 (3.5)
Nicotine	172 (44.6)	165 (37.9)
Others	9 (2.3)	7 (1.6)
Psychiatric History^a^		
Anxiety	144 (37.3)	170 (39.1)
PTSD	70 (18.1)	75 (17.2)
Depression	149 (38.6)	158 (36.3)
Psychotic	9 (2.3)	9 (2.1)
Bipolar Behavior	56 (14.5)	51 (11.7)
Behavior, Conduct Disorder^b^	49 (12.7)	57 (13.1)
Borderline Personality Disorder	7 (1.8)	8 (1.8)
Other comorbidity	33 (8.6)^c^	18 (4.1)

a Missing cases for drug use disorder (n = 1–13), psychiatric history (n = 8–13) were not shown at Table 1.

b Including oppositional defiant, Intermittent explosive.

c Differences in two groups at p < 0.01.

### Analysis

To test the non-inferiority of 6-month retention between post-COVID intakes (exposure group) and control group, a multivariable logistic regression model was fit that included an indicator term for group as well as fixed effect terms for relevant patient-level covariates. Using a Farrington-Manning test, the margin of inferiority was set at 0.05 with alpha = 0.1 and an 80% confidence interval.

Selection for covariates that may influence retention was restricted to basic demographics and otherwise parsimonious based on prior published studies’ findings of oft-significant baseline characteristics to enhance power. The odds ratio for the group term was estimated along with a two-sided 95% confidence interval. Additionally, a site indicator was treated as a fixed effect in logistic models. As a secondary analysis, an adjusted Cox proportional hazards model was estimated for time to discontinuation.

To test non-inferiority for secondary outcomes, adjusted generalized linear models were fit, using the appropriate link function for each outcome (logit-link for binary outcomes). Using the same methodology, each effect estimate was compared with the null value plus or minus the margin of inferiority (depending on which direction was considered beneficial for each outcome).

Finally, to investigate moderation of the association between groups and each outcome by baseline variables, a moderator∗group term was added in a separate model for each potential moderator. When significant, separate effect estimates and their corresponding 95% confidence intervals were calculated and reported for each categorical level of the moderator. An additional analysis tested for potential site-level variation in the impact of the pandemic regulatory change on retention. Anticipating variation across the 9 sites in take-home frequencies, especially in the post-COVID period, we analyzed the median increase in the number of days per month that were take-home doses and split sites into either loose or restrictive groupings. All analyses were conducted in SAS 9.4.

### Role of the funding sources

This study was funded by the US NIDA CTN (USDHHS NIDA CTN UG1 DA013035-15). The CTN approved the protocol and manuscript before the study commenced and before submission to the journal. Funders had no role in study design, data collection, data analysis, interpretation, writing of the report or decision to submit.

## Results

Among 24 applicant OTPs through the CTN, funding allowed for the selection of 10 OTP sites. One site subsequent to selection could not complete data collection due to state law requiring advanced informed consent from patients in OTPs. Therefore, across the fully participating 9 OTPs, a total of 435 individuals were admitted in the post-COVID period and 386 admitted in the year-ago control period for a total sample of 821 individuals. Included patients had an average age of 38.3 (SD 11.1) and 58.9% were male (Table 1). There were no statistical differences in the distribution of individuals’ demographic characteristics, justice involvement, infectious disease status, addiction severity, and comorbidities between the two groups, with few exceptions (Table 1, Supplementary Table S1). The only significant difference was a higher prevalence of psychostimulant use disorder (cocaine or methamphetamine) among patients in the post-COVID onset period compared to controls (32.9% vs 25.7%, *X*^*2*^ (1) = 5.32, p = 0.02).

Separate bivariate analyses were conducted to test the associations between baseline characteristics and six-month retention using Wald's chi-square tests in logistic regression models. Baseline characteristics with an overall *X*^*2*^ statistic associated with a p-value <0.05 in the univariate models were then entered into a multivariable logistic regression model (Table 2). Retention rates among patients admitted post-COVID were not different from those admitted pre-COVID (60.0% vs 60.1%) when adjusting for site. The noninferiority Farrington-Manning test showed that post-COVID 6-month retention was not inferior to pre-COVID with a margin = 0.05, alpha = 0.1, and 80% confidence interval (−0.043, 0.041).

**Table 2:** Six month retention in treatment comparing new intakes Pre- and Post-COVID onset (N = 821).

	No (%)	Adjusted Odds Ratio (95% CI)^a^
Treatment period		
Pre-COVID	232/386 (60)	Reference
Post-COVID	261/435 (60)	1.00 (0.74–1.37)
Age		
<40	266/485 (55)^d^	Reference
≥40	227/336 (68)	1.42 (1.02–1.96)^c^
Gender		
Male	307/492 (62)	Reference
Female	186/329 (57)	0.96 (0.70–1.31)
Race		
White	366/608 (60)^c^	Reference
African American	60/85 (71)	0.96 (0.53–1.72)
Asian, Native Hawaiian or Pacific Islander, American Indian or Alaskan Native	11/30 (37)	0.39 (0.15–0.98)
Other	43/64 (67)	1.00 (0.45–2.22)
Missing	13/34 (38)	0.47 (0.21–1.03)
Ethnicity		
Hispanic	55/83 (66)	1.03 (0.51–2.07)
Non-Hispanic	351/585 (60)	Reference
Missing	87/153 (57)	0.85 (0.42–1.71)
Marital Status		
Never married	152/297 (51)	
Married	119/184 (65)	
Formerly married	89/141 (63)	
Single (never, ever married)	127/185 (69)	
Missing	6/14 (43)	
Education		
<8 years	22/39 (56)	
Some high school	111/189 (59)	
High School graduate	196/317 (62)	
Some college	112/180 (62)	
College graduate	37/69 (54)	
Missing	15/27 (56)	
Housing^b^		
Secure housing	312/48 (65)^e^	Reference
Homeless	55/123 (45)	0.60 (0.44–0.83)^d^
Unstable (transiently housed family/friends)	103/174 (59)	
Other	14/29 (48)	
Missing	9/15 (60)	
Employment^b^		
Unemployed (looking a job)	159/301 (53)^c^	0.73 (0.53–1.01)
Out of labor force (unemployed, not looking)	104/175 (59)	Reference
Retired, Disabled, Student	72/102 (71)	
Full-time	100/155 (65)	
Part-time	49/74 (66)	
Missing	9/14 (64)	
Drug use disorder		
Cannabis	49/100 (49)	
No cannabis use disorder	437/710 (62)	
Cocaine	73/119 (61)	
No cocaine use disorder	416/696 (60)	
Stimulant	54/140 (39)^d^	0.58 (0.35–0.98)^c^
No stimulant use disorder	435/673 (65)	Reference
Onset opioids		
Aged <18	144/249 (58)	
Aged 18–25	165/276 (60)	
Aged >25	165/262 (63)	
Missing	19/34 (56)	
Route for opioids		
Injection	259/474 (55)	
Oral	32/50 (64)	
Smoking	9/20 (45)	
Intranasal	173/246 (70)	
Missing	20/31 (65)	

a Site was controlled in all models. Age, gender, race, ethnicity and significant covariates in the univariate model were included in the multivariable model. One dummy variable for 36 patients those who missing data on housing (n = 15), employment (n = 14), or drug use disorder (n = 8) was included and non-significant in the multivariable model (*X*^*2*^ (1) = 0.0005, p = 0.98).

b Retention rates were significantly different between the cases with and without secure housing and the cases between unemployed and not unemployed, so binary group was used in the multivariable model.

c Differences among groups at p < 0.05.

d p < 0.01.

e p < 0.001.

**Fig. 2:**
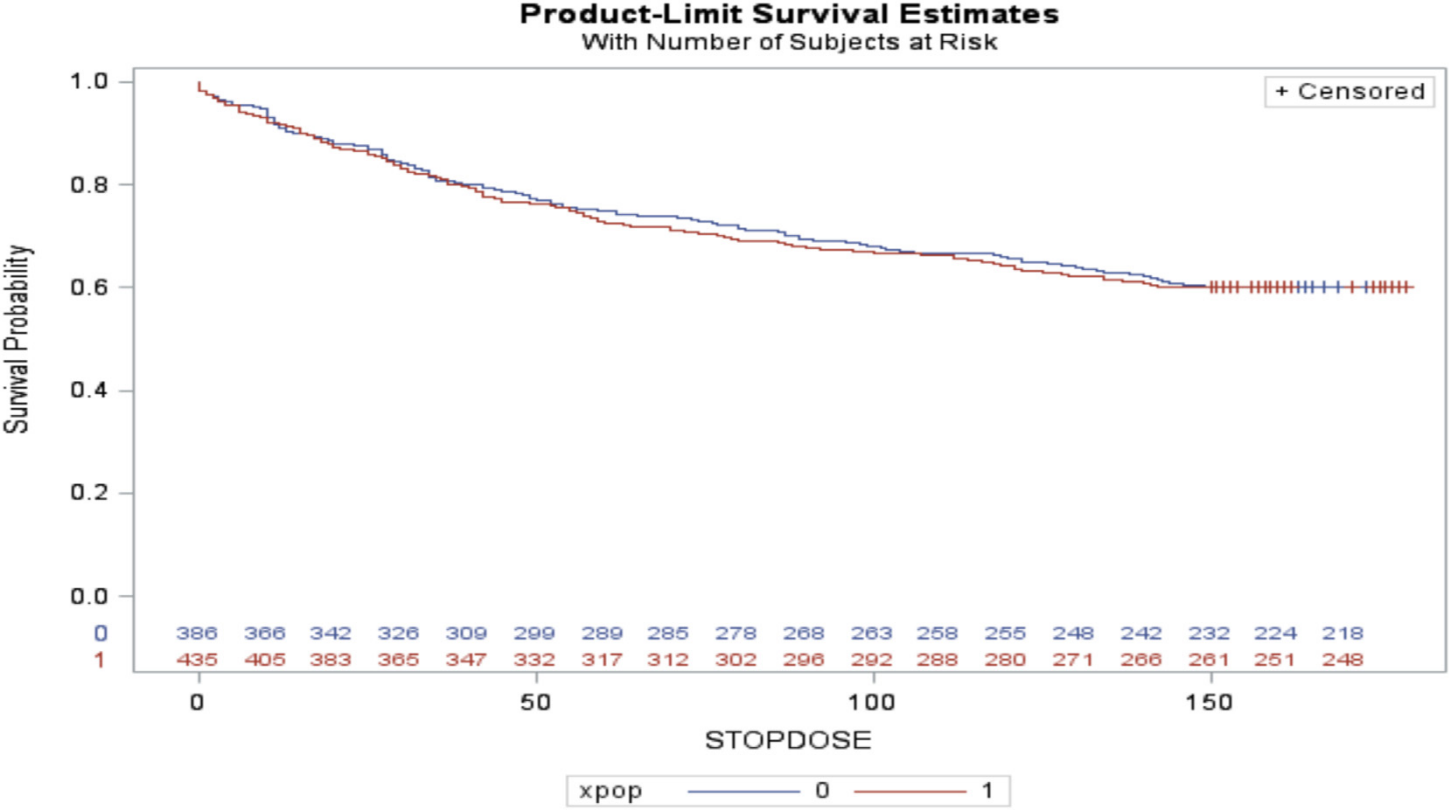
**Treatment retention survival curves between Pre-COVID-19 and Post-COVID-19 (N = 821)**. Survival curves did not significantly differ before-COVID-19 and post-COVID-19 by using Log–Rank test [*X*^*2*^ (1) = 0.02, p = 0.8857].

Time to discontinuation was also non-inferior between groups: survival curves did not significantly differ before-COVID-19 and post-COVID-19 by using Log–Rank test [*X*^*2*^ (1) = 0.02, p = 0.8857] (Fig. 2). In the Cox's proportional hazard model, controlling for site, age, gender, race, ethnicity, secure housing, unemployment status and stimulant use at baseline, the hazard ratio for time to discontinuation for post-COVID-19 group was not significantly different than from the before-COVID period (HR = 1.02, 95% CI = 0.81–1.27). Secondary analyses regarding cause of discontinuation did not find significant variation in documented reasons for discontinuation between the pre-COVID and post-COVID periods, with the most common reasons being lost to follow-up and leaving against advice (Supplementary Table S2).

Gray's Test for Equality of Cumulative Incidence Functions determined there were no differences between the two groups in rates of adverse events in the aggregate which included ED visits, hospitalizations, and overdose (*X*^*2*^ (1) = 0.55, p = 0.46) (Table 3, Supplementary Table S3). In adjusted analyses with a test for non-inferiority, rates of adverse events in post-COVID 6 months were not inferior to pre-COVID with a margin = 0.05, alpha = 0.1, and 80% confidence interval (−0.057, 0.007).

**Table 3:** Adverse events in CTN012 in the multivariable models (N = 821).

	With 1+ AE^a^	With 1+ ED visit	With 1+ Hospitalization
	Odds (95% CI)	Odds (95% CI)	Odds (95% CI)
Treatment period			
Pre-COVID	Ref.	Ref.	Ref.
Post-COVID	0.81 (0.54–1.22)	0.91 (0.49–1.67)	0.78 (0.47–1.29)
Age			
<40	Ref.	Ref.	Ref.
≥40	0.57 (0.36–0.90)^b^	0.72 (0.36–1.44)	0.59 (0.33–1.07)
Gender			
Male	Ref.	Ref.	Ref.
Female	1.00 (0.66–1.53)	1.05 (0.54–2.03)	1.44 (0.85–2.44)
Race			
White	Ref.	Ref.	Ref.
African American	1.31 (0.62–2.78)	1.81 (0.59–5.57)	1.64 (0.69–3.91)
Asian, Native Hawaiian or Pacific Islander, American Indian or Alaskan Native	1.50 (0.52–4.30)	2.13 (0.57–8.03)	2.58 (0.78–8.54)
Other	1.19 (0.44–3.21)	1.32 (0.30–5.75)	1.39 (0.46–4.21)
Ethnicity			
Hispanic	Ref.	Ref.	Ref.
Non-Hispanic	0.93 (0.40–2.17)	0.88 (0.27–2.91)	0.66 (0.26–1.71)
Missing	0.39 (0.13–1.15)	0.25 (0.06–1.00)	0.51 (0.14–1.81)
Housing			
Secure housing	Ref.		Ref.
Homeless/unstable/other/missing	2.16 (1.43–3.26)^d^		2.32 (1.37–3.93)^c^

Ref. = Reference group.

a Adverse events include overdose, ED visit, Inpatient hospitalization, intoxication/altered mental status, criminal justice involvement.

b Differences among groups at p < 0.05.

c p < 0.01.

d p < 0.001.

While there was no difference in rates of adverse events between groups, we did find significantly higher rates of opioid use at all tested time points among patients in the post-COVID cohort compared to pre-COVID controls (64.8% v 51.1%, p < 0.001) (Supplementary Table S4). Across both time periods, a total of 100 (12.2%) patients did not have a single documented urine drug test at any point during care (41 (10.6%) before COVID and 59 (13.6%) after COVID) and were excluded from toxicology analyses. Multivariate analyses found no significant associations between patient characteristics and rates of opioid use with the exception of preferred route of opioid administration, in which injection route was associated with greater opioid use than oral administration (aOR 4.98, 95% CI 2.17–11.41).

Initial findings led to restricting moderator analyses to a single baseline characteristic, the presence of a comorbid stimulant use disorder at intake. A moderator∗group term for comorbid stimulant use disorder was added in a separate model and did not identify significant differences in outcomes including retention, adverse events, and opioid use among those with and without comorbid stimulant use (Supplementary Table S3).

Finally, an additional analysis tested for site-level variation in the impact of the pandemic regulatory change on retention. Implementation of take-home recommendations varied greatly across the 9 sites, especially in the post-COVID period. Across all sites, the median increase in the number of days per month that were take-home dosing days increased by 3 days (Supplementary Figure S1). Four of the 9 sites had higher rates of take-homes in the post-COVID period than the median split and were grouped into a “less restrictive” category (Supplementary Figure S1). The other five sites that increased monthly take-homes by fewer than 3 days were grouped into a “more restrictive” category. An analysis investigating the possible moderating effects of less versus more restrictive increases in take-home dosing on study outcomes found that there were no differences across group effects in retention, adverse events, or opioid use outcomes comparing the pre-COVID and post-COVID cohorts (Supplementary Table S5). Consistent with the primary analysis, rates of opioid use were elevated in post-COVID periods irrespective of group classification.

## Discussion

We found that treatment outcomes post-COVID under relaxed regulations were non-inferior to those of the year-ago control group despite higher rates of opioid use. Our findings suggest that allowing for up to 14 and 28 days of take-homes early in care may not be associated with increased dropout or adverse events. This is consistent with pre-COVID studies that showed no differences in retention with less frequent in-person MOUD dosing,^23^^,^^24^ but warrants further research as findings should be interpreted with caution, especially as COVID-era disruptions to ED and inpatient service utilization may have confounded rates of adverse events characterised in the post-COVID period. A potential result might be the under-use of acute care services due to avoidance and concerns about COVID exposure rather than a true decrease in the need for ED or hospital-based care.

There was variation in allowances for take-home doses among the 9 sites. While roughly half of the sites kept similar schedules of take-homes for new intakes in the post-COVID period compared to the pre-COVID period, the other half significantly liberalized numbers of take-home doses. While descriptive analyses grouping sites by loose and restrictive policies did not affect findings, future research could further assess clinic- and patient-level characteristics that might be associated with take-home schedules and subsequent outcomes.

In recent decades there have been a few lines of research, some in other countries, suggesting that lower threshold methadone maintenance treatment may be non-inferior to programs that require more frequent visits, more intensive group and individual sessions, and more frequent drug testing.^25^, ^26^, ^27^, ^28^ Much of this research has examined relatively lower-intensity counselling requirements and suggested that it is non-inferior to high-intensity treatment, whether for buprenorphine or methadone.^25^, ^26^, ^27^, ^28^ A few studies have suggested that less frequent clinic visits for observed dosing with more generous take-home schedules, which substantially reduce participant burdens, are non-inferior to more frequent visit requirements.^9^^,^^29^^,^^30^ These findings have largely been echoed in recent post-COVID qualitative studies finding patients feel more liberated and accomplished when having more control over their treatment and that reduced visit frequency allows for more time for other important activities in life.^9^^,^^31^ However, at least one study, in Italy, found that less frequent take-homes may be associated with worse drug use outcomes if there are no consequences (contingencies) in response to drug-positive urines,^30^ also consistent with some post-COVID surveys of OTP clinicians and administrators regarding possible increased risks with less frequent visits.^21^ More recently, there have been a handful of post-COVID studies evaluating the impact of extended take-homes and regulatory reforms on outcomes for patients receiving methadone maintenance treatment.^31^, ^32^, ^33^, ^34^, ^35^ Primarily these have been qualitative surveys of patient or provider attitudes and experiences,^31^ empirical studies of visit frequency but not clinical correlates,^32^ and limited to single sites or regions.^33^, ^34^, ^35^ This is the first study with patient-level EHR data for an exposed group analyzed in comparison with a year-ago control group across multiple sites. While complimentary to previous qualitative studies, mixed-methods approaches to understanding the effects of extended take-home policies may aide in interpreting EHR-based analyses. While we chose 6-month retention as the primary outcome, there is variation across OUD treatment studies in which outcomes to prioritize related to patterns of drug use, treatment adherence, and adverse events.^36^^,^^37^ Further, disparities in outcomes were uncovered, in particular lower 6-month retention rates (aOR 0.39 (0.15 to 0.98)) among Asian, Native Hawaiian or Pacific Islander, American Indian or Alaskan Native populations that warrant further investigative work to determine which interventions may aide in clinical outcomes.

Limitations to our study are common to those with retrospective observational data. In particular, we cannot account for unmeasured confounders that may have affected outcomes. Specifically given the study period, we could not isolate the impact of extended take-homes from other clinic policies. Further, we could not account for societal or financial disruptions due to COVID-19 lockdowns and safeguards. Relatedly, EHR data were unable to systematically catalog other outcomes of interest such as quality of life or level of functioning that may have been impacted by less burdensome visit requirements. Two salient variables excluded from study analyses were patterns of methadone dosing and service utilization which warrant further research. Additionally, methadone diversion was not an outcome of the study as it would not typically be well characterized in EHR data in a complete and structured manner reliably across sites. Regarding external generalizability related to site selection and the patient case mix attending each site, OTPs were recruited through the NIDA CTN network, first established over 20 years ago.^13^ Therefore, there was an overrepresentation of academically affiliated non-profit OTPs with prior NIH-funded research experience. These sites have different patient caseloads, policies, and clinical outcomes, especially compared to private for-profit OTPs which have proliferated in the past 10–15 years of the opioid crisis. Finally, while quality assurance mechanisms were in place throughout the study, there may have been human error in data entry although there is a low likelihood it would have been systematic in a way to change our findings. Compared to other EHR record systems, a strength of OTP EHR archives is that federal law requires rigorous and complete documentation of dosing schedules given that methadone is a Schedule II controlled substance.

In conclusion, this is the first multi-site empirical study of patient outcomes under the SAMHSA blanket waiver allowing for extended take-homes following COVID onset that uses a control group. Overall, we identified relatively modest increases in take-home schedules for patients newly beginning treatment. We found patients had equivalent retention in care at 6 months and equivalent risk of adverse events while in care despite slightly higher rates of opioid use at the group level, even among sites with routine take-homes exceeding 7–14 days early in treatment. These findings are meaningful for clinical practice as well as ongoing debates about policy reform as the COVID-19 era public health emergency winds down over the course of 2023–2024. Based on these findings, randomized trial methodologies, likely incorporating novel trial designs, could be conducted to confirm the non-inferiority of extended take-homes and reduced visits for successfully retaining patients in care while monitoring patterns of drug use and adverse events.

## Contributors

ARW, NK, MC, JR, EVN: literature search, figures, study design, data interpretation, writing.

MH: figures, data collection, data analysis, data interpretation, writing.

LH, NA: data collection, data analysis, data interpretation, writing.

Both NK and MH had direct access to the data.

## Data sharing statement

Data will be made publicly available on the NIDA CTN website.

## Declaration of interests

ARW receives consulting fees, equity, and travel expenses for a leadership role at Ophelia Health, Inc. a telehealth platform for the treatment of opioid use disorder. NK receives consulting fees for ongoing service as an expert witness in opioid litigation. TR has served as Principal Investigator and co-investigator on studies for which support in the form of donated or discounted medication, smartphone apps, and/or funds was provided by Alkermes, Inc.; Indivior, Inc.; Braeburn Pharmaceuticals, Inc.; Pear Therapeutics; CHESS Health, and Data Cubed. None of this support has gone directly to the investigators, to NYU, to NIDA/NIH, or to NIDA's contractor Emmes, Inc. All other authors have no interest to disclose.
