# Supplementary material for: Retention and critical outcomes among new methadone maintenance patients following extended take-home reforms: a retrospective observational cohort study
Source: Lancet Reg Health Am. 2023 Dec 4;28:100636. doi: 10.1016/j.lana.2023.100636 (PMC10751716; doi:10.1016/j.lana.2023.100636)
Supplement: Supplementary Tables [file mmc1.pdf]

Supplementary Tables

Manuscript: Retention and Critical Outcomes among New Methadone Maintenance Patients Following Extended Take-Home Reforms: A Retrospective Observational Cohort Study

Authors: Arthur Robin Williams, MD, MBE; Noa Krawczyk, PhD; Mei-Chen Hu, PhD; Lexa Harpel, BA; Nicole Aydinoglo, MHA; Magdalena Cerda, DrPH; John Rotrosen, MD; Edward V Nunes, MD

***Correspondence to:** Arthur Robin Williams, MD, MBE
Assistant Professor of Clinical Psychiatry, Columbia University Department of Psychiatry.
Research Scientist II, New York State Psychiatric Institute
Office phone: +1 347-857-8015
E-mail: aw2879@cumc.columbia.edu

Table S1: History of Substance Use of New Intakes at Opioid Treatment Programs, by COVID-19 Exposure (N=821)

	**Pre-COVID (n=386)** **No. (%)/Mean (SD)**	**Post-COVID (n=435)** **No. (%)/Mean (SD)**
Primary Drug of Choice		
Opioids	384 (99.5)	430 (98.9)
Cannabis	0 (0.00)	1 (0.2)
Methamphetamine	0 (0.00)	2 (0.5)
Other	0 (0.00)	1 (0.2)
Missing	2 (0.5)	1 (0.2)
Route for primary drug		
Oral	29 (7.9)	22 (5.2)
Smoking	8 (2.2)	13 (3.1)
Intranasal	119 (32.3)	127(30.2)
Injection	211 (57.5)	261(61.6)
Onset age of primary drug	24.7 (10.1)	24.0 (8.9)
Secondary Drug of Choice		
Opioids	37 (9.6)	34 (7.8)
Alcohol	23 (6.0)	25 (5.7)
Cocaine	92 (23.8)	106 (24.4)
Cannabis	60 (15.5)	61 (14.0)
Amphetamine	1 (0.3)	3 (0.7)
Methamphetamine	46 (11.9)	63 (14.5)
Benzodiazepines	14 (3.6)	23 (5.3)
Other	20 (5.2)	13 (3.0)
None	65 (16.8)	86 (19.8)
Missing	28 (7.3)	21 (4.8)
Route for Secondary Drug		
Oral	48 (18.8)	50 (17.4)
Smoking	117 (45.9)	131 (45.5)
Intranasal	33 (12.9)	40 (13.9)
Injection	56 (22.4)	67 (23.3)
Onset age of secondary drug	21.8 (9.2)	20.9 (9.0)
Third Drug of Choice		
Opioids	6 (1.5)	3 (0.7)
Alcohol	19 (4.9)	26 (6.0)
Cocaine	19 (4.9)	21 (4.8)
Cannabis	40 (10.4)	52 (11.9)
Hallucinogens	1 (0.3)	0 (0.0)
PCP	1 (0.3)	0 (0.0)
Amphetamine	2 (0.5)	6 (1.4)
Methamphetamine	8 (2.1)	12 (2.8)
Benzodiazepines	14 (3.6)	16 (3.7)
Other	20 (5.2)	27 (6.2)
None	189 (49.0)	212 (48.7)
Missing	67 (17.4)	60 (13.8)
Route for Third Drug		
Oral	36 (33.0)	44 (36.4)
Smoking	56 (51.4)	57 (47.1)
Intranasal	8 (7.3)	9 (7.4)
Injection	9 (8.3)	11 (9.1)
Onset age of third drug	18.1 (8.8)	16.5 (7.0)
Tobacco use		
Yes	312 (80.8)	358 (82.3)
No	58 (15.0)	52 (11.7)
Missing	16 (4.2)	26 (6.0)

Table S2: Treatment Discontinuation by Cause (N=328)

	**Pre-COVID (N=154) No. (%)**	**Post-COVID (N=174) No. (%)**	**Difference[a] $X^2(1)$, p**
Discharged			
Completed program	2 (1.3)	1 (0.6)	
Transferred to another program	17 (11.0)	22 (12.6)	0.17, 0.4576
Left against advice	59 (38.3)	55 (31.6)	0.99, 0.3208
Lost to follow-up	41 (26.6)	68 (39.1)	3.38, 0.0661
Behavioral disruption	1 (0.7)	0 (0)	
Other	7 (4.6)	3 (1.7)	
Imprisoned	9 (5.8)	5 (2.9)	
Hospitalized/higher level of care	0 (0)	2 (1.2)	
Died	2 (1.3)	1 (0.6)	
Missing	16 (10.4)	18 (10.3)	

[a] Multivariable models controlled for site, age, gender, race, ethnicity for all three reasons for discontinuation indicated; one additional variable secure housing was also controlled for in the model on “left against advice”.

Table S3: Outcomes among Pre-Covid and Post-Covid Intakes, by baseline Stimulant Use Disorder (N=813[a])

	Pre-COVID No. (%)	Post-COVID No. (%)	Group Differences by Stimulant Use $X^2(1)$ or F, p
**6-month retention**			
Simulant use disorder	26/58 (45)	28/82 (34)	$X^2(1) = 2.86$,
No Simulant use disorder	205/326 (63)	230/347 (66)	p = 0.0910
**Adverse Events**[b]			
Having 1+ adverse event			
Simulant use disorder	14/58 (24)	15/82 (18)	$X^2(1) = 0.49$,
No Simulant use disorder	56/326 (17)	58/347 (17)	p = 0.4821
ED visit			
Simulant use disorder	6/58 (10)	8/82 (10)	$X^2(1) = 0.16$,
No Simulant use disorder	21/326 (6)	23/347 (7)	p = 0.6852
Inpatient hospitalization			
Simulant use disorder	8/58 (14)	6/82 (7)	$X^2(1)=,1.29$
No Simulant use disorder	31/326 (10)	33/347 (10)	p = 0.2559
**Opioid use**[c]			
Simulant use disorder	97/291 (48)	149/241 (62)	F(1, 2122) = 0.00,
No Simulant use disorder	625/1207 (52)	790/1192 (66)	p = 0.9829

[a] 8 patients missing baseline stimulant use disorder were excluded, and site was controlled.
[b] Patients in the site 9 were not included, due to absence of adverse event data
[c] Rates of opioid use reflect averaged monthly rates of opioid use in care across groups

Table S4: Percent of patients testing positive for opioids on a monthly basis following intake of methadone maintenance treatment (N=721[a])

Month	Pre-COVID (n=345)	Post-COVID (n=376)	Differences between two groups	
	No. positive /observed patients[b] (%)	No. positive /observed patients[b] (%)	X^2	p-value
1	181/301 (60.1)	215/296 (72.6)	$X^2(1) = 10.45$	0.0012
2	149/265 (56.2)	177/265 (66.8)	$X^2(1) = 6.24$	0.0124
3	114/240 (47.5)	152/240 (68.3)	$X^2(1) = 12.18$	0.0005
4	96/219 (43.8)	139/226 (61.5)	$X^2(1) = 13.93$	0.0002
5	100/210 (47.6)	130/217 (59.9)	$X^2(1) = 6.49$	0.0109
6	84/181 (46.4)	127/206 (61.7)	$X^2(1) = 9.03$	0.0027
All	724/1416 (51.1)	940/1450 (64.8)	$F(1, 2140) = 19.66$	<.0001

[a]100 patients without any UDS results (41 (10.6) pre-COVID and 59 (13.6) post-COVID) at any point during care were excluded.
[b]Positive refers to patients with one or more positive urine test results performed in a given month.

Table S5: Outcomes among Pre-Covid and Post-Covid Intakes, Grouping OTP Sites by Loose and Restrictive[a] Changes in Take-home Schedules (N=821)

	Pre-COVID No. (%)	Post-COVID No. (%)	Group Differences by site $X^2(1)$ or F, p
**6-month retention**			
Loose take-home scheduling	109/171(64)	110/192 (57)	$X^2(1) = 2.72$, $p = 0.0990$
Restrictive take-home scheduling	123/215 (57)	151/243 (62)	
**Adverse Events**			
Patients with 1+ adverse event			
Loose take-home scheduling	47/171 (27)	44/192 (23)	$X^2(1) = 0.70$, $p = 0.4028$
Restrictive take-home scheduling	24/215 (11)	29/243 (12)	
Patients with 1+ ED visit			
Loose take-home scheduling	24/171 (14)	24/192 (13)	$X^2(1) = 0.68$, $p = 0.4102$
Restrictive take-home scheduling	4/215 (2)	7/243 (3)	
Patients with 1+ Inpatient hospitalization			
Loose take-home scheduling	26/171 (15)	22/192 (11)	$X^2(1) = 0.68$, $p = 0.4071$
Restrictive take-home scheduling	14/215 (7)	17/243 (7)	
**Opioid use**[b]			
Loose take-home scheduling	332/639 (52)	415/622 (67)	$F(1, 2140) = 0.00$, $p = 0.9722$
Restrictive take-home scheduling	392/777 (50)	525/828 (63)	

[a] Loose= Sites above the median split (with 3+ take-home days per month), Restrictive= Sites below the median split (with 0-2 take-home days per month)

[b] Rates of opioid use reflect averaged monthly rates of opioid use in care across groups
