# Supplementary material for: Retention and critical outcomes among new methadone maintenance patients following extended take-home reforms: a retrospective observational cohort study
Source: Lancet Reg Health Am. 2023 Dec 4;28:100636. doi: 10.1016/j.lana.2023.100636 (PMC10751716; doi:10.1016/j.lana.2023.100636)

eFigure 1: Methadone Dosing among New Intakes

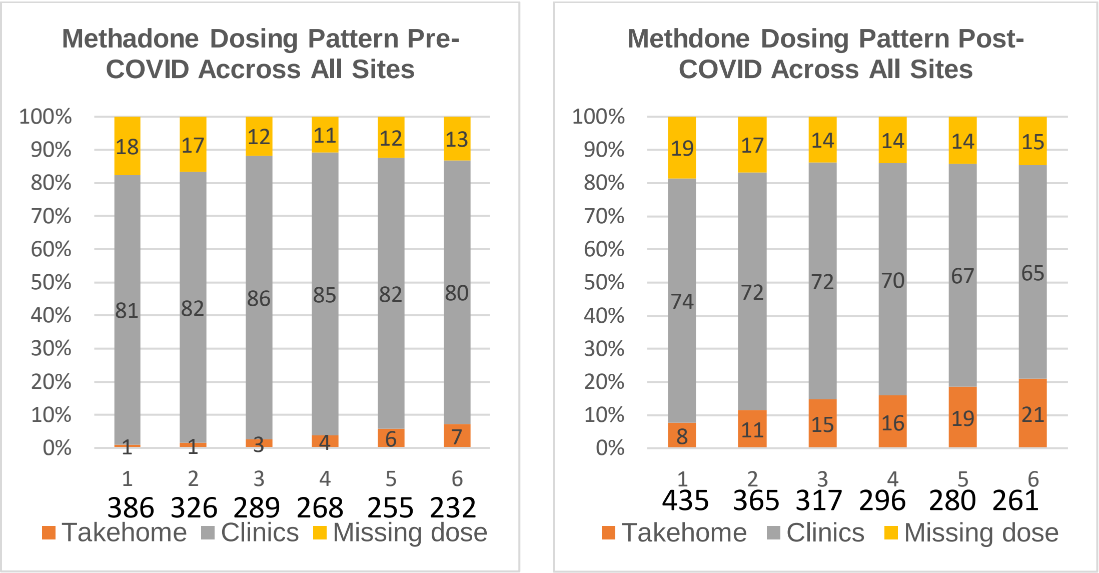

a. Restrictive take-home scheduling: Sites below the median split (with an increase of 0-2 take-home days per month compared to pre-COVID period)

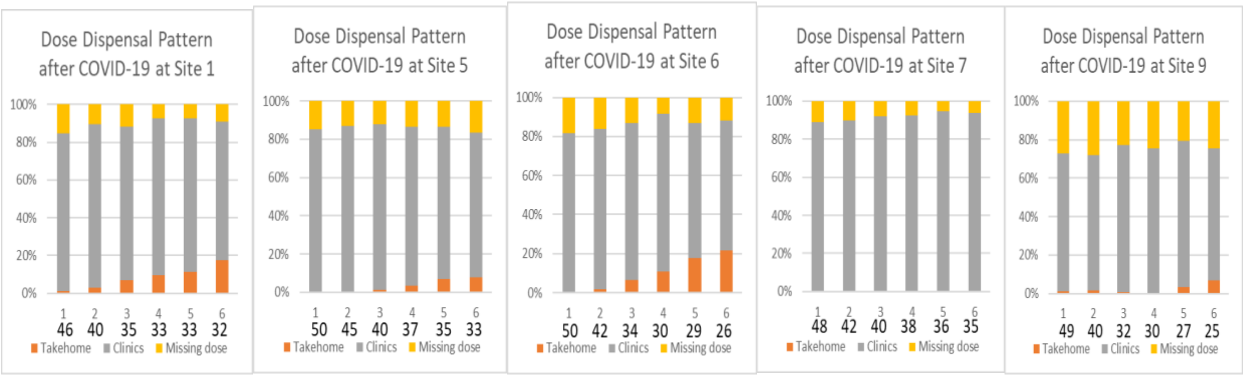

b. Loose take-home scheduling: Sites above the median split (with 3+ additional take-home days per month compared to pre-COVID period)

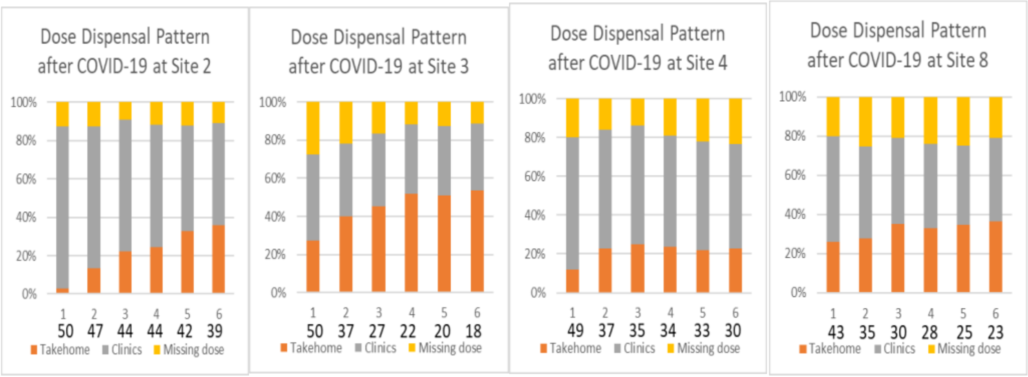

Supplement: Supplementary Figure [file mmc2.pdf]
